# Management of Placental Abruption Following Blunt Abdominal Trauma

**DOI:** 10.7759/cureus.10337

**Published:** 2020-09-09

**Authors:** Nolan Page, Kristina Roloff, Arnav P Modi, Fanglong Dong, Michael M Neeki

**Affiliations:** 1 Emergency Medicine, Arrowhead Regional Medical Center, Colton, USA; 2 Emergency Medicine, California University of Science and Medicine, San Bernardino, USA; 3 Obstetrics and Gynecology, Arrowhead Regional Medical Center, Colton, USA; 4 Obstetrics and Gynecology, California University of Science and Medicine, San Bernardino, USA

**Keywords:** trauma, pregnancy, placental abruption

## Abstract

Blunt abdominal trauma during pregnancy poses a significant risk to both the mother and fetus. Here, we review a case of a 21-year-old female at 17 weeks' gestation involved in a motor vehicle accident, who subsequently suffered a placental abruption and fetal demise secondary to the trauma. We present a review of traumatic placental abruptions, including epidemiology, laboratory findings, imaging, and management strategies. Because of associated maternal and fetal morbidity and mortality, it is imperative that health care professionals are well versed in the diagnosis, treatment, and acute care for this rare, yet high-risk scenario.

## Introduction

Trauma is the leading cause of non-obstetric maternal death. Roughly 8% of pregnancies experience some form of trauma in the United States. In developed countries, motor vehicle collisions (MVCs) are the leading cause of obstetric trauma and account for up to 80% of trauma in pregnancy; other major causes include falls, assaults, and domestic violence [1-4].

Placental abruption accounts for about 1% of complications in pregnancies. Placental abruption extending more than 50% of the placenta is associated with a higher likelihood of fetal demise [5]. Although normally associated with painful vaginal bleeding in the second or third trimester, placental abruption following a traumatic event can occur without significant vaginal bleeding or regular uterine contractions. In addition, coagulopathy, maternal hemorrhage, and fetal death are potential and serious complications of placental abruption [2,5,6].

The management of trauma in pregnancy is similar to that in a non-pregnant patient, and begins at the first point of contact with the victim - whether in the field at the location of the accident, or in the emergency department when the patient presents. However, trauma algorithms must deviate towards obstetric care when pregnancy-specific complications such as placental abruption occur due to trauma. Here, we present a case of placental abruption following blunt abdominal trauma and focus on the approach to optimal care in regard to placental abruption relating to trauma during pregnancy.

## Case presentation

A 21-year-old G2P1 patient with no past medical history presented to the emergency department at 17 1/7 weeks' gestation complaining of lower abdominal cramping and vaginal bleeding following an MVC earlier the same day. The patient was a restrained driver-side rear passenger involved in a moderate speed MVC in which her vehicle was struck on the rear passenger side at approximately 40 miles per hour. Side curtain airbags were deployed. She was able to extricate herself from the vehicle with no difficulty and was ambulatory. She had complaints of minimal abdominal pain without vaginal bleeding. She was evaluated at the scene of the accident by emergency medical services and advised to seek immediate medical attention. She declined transport against medical advice and was told to seek care if her condition changed.

An hour after the collision, while at home, the patient experienced worsening abdominal pain and developed vaginal bleeding (approximately 20 mL). At this point, she activated Emergency Medical Services (EMS) and was transported to the closest trauma center. On arrival to the trauma center, she was immediately assessed by the trauma and obstetrics teams. Her vital signs included a blood pressure of 133/77 mmHg, heart rate of 105 beats per minute (bpm), respiratory rate of 18 breaths per minute, and oxygen saturation of 98% on room air. Physical examination revealed no evidence of distress, and a gravid abdomen with minimal tenderness to palpation in the lower abdomen.

**Figure 1:**
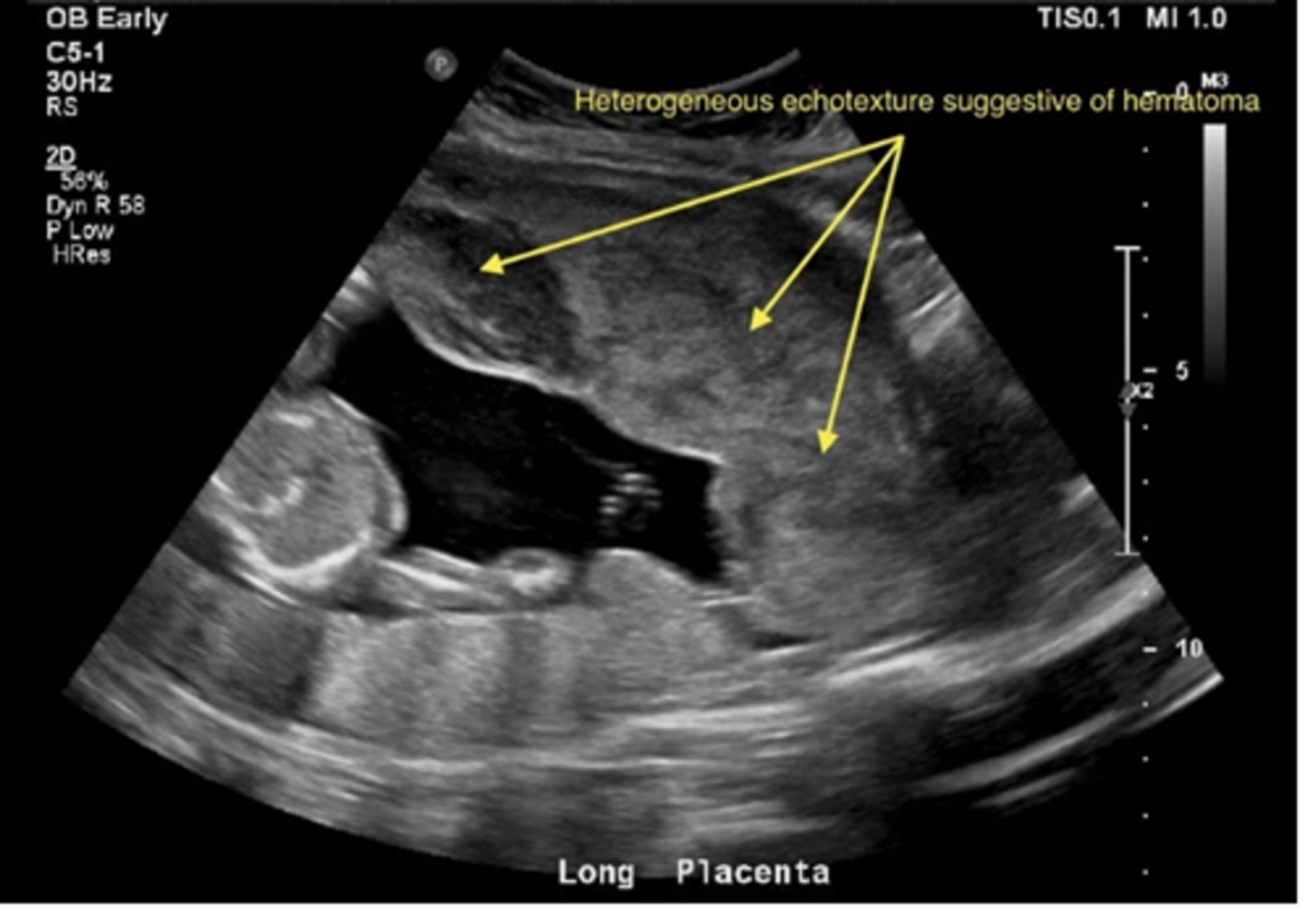
"U/S OB trauma limited" post motor vehicle collision, showing notable posterior placenta with a heterogeneous echotexture structure extending anteriorly U/S, ultrasound; OB, obstetric; findings were concerning for placental abruption.

**Figure 2:**
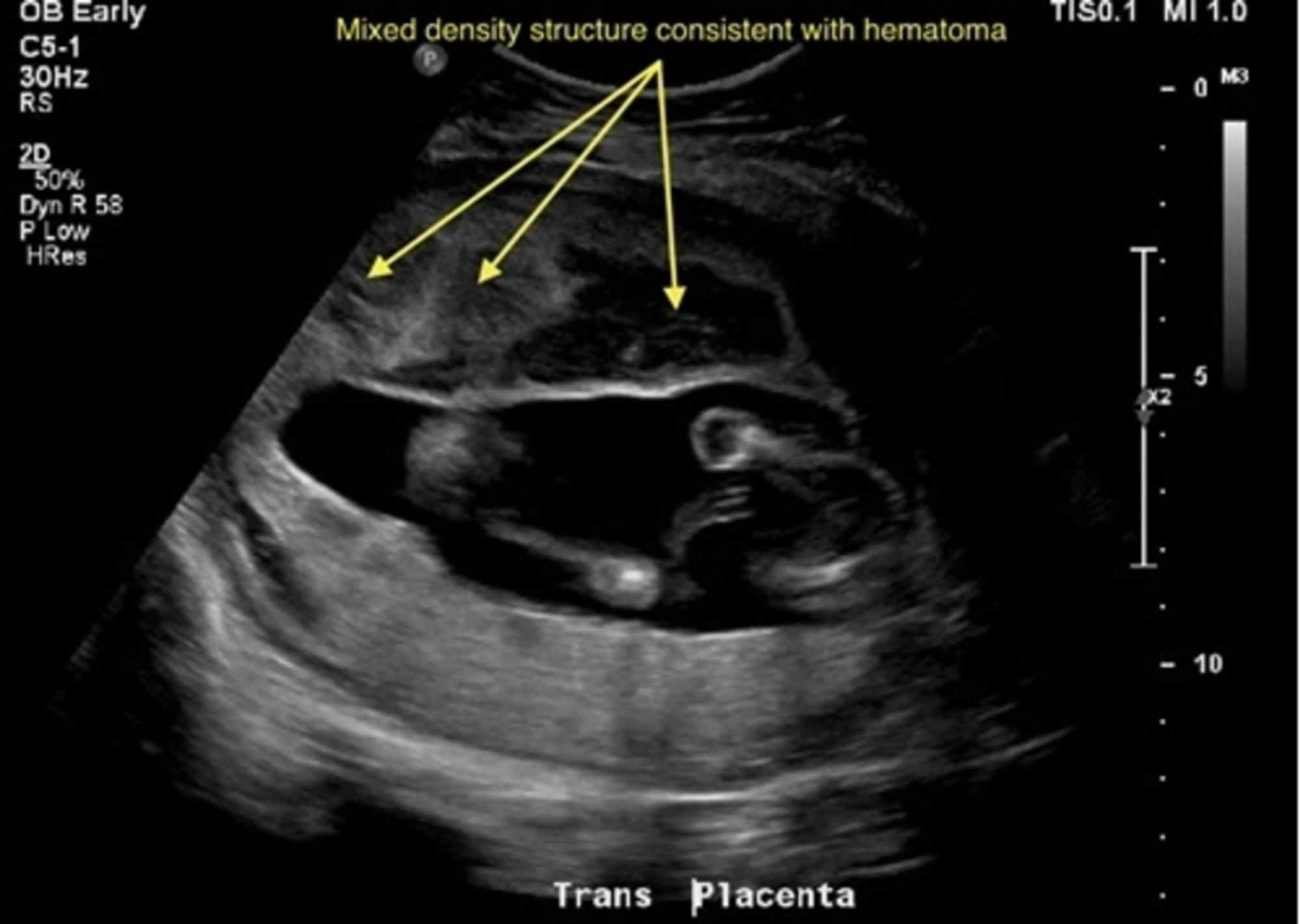
"U/S OB trauma limited" post motor vehicle collision, showing notable anterior echogenic material, which appears heterogeneous in echotexture with hypoechoic areas measuring 12.0 x 5.4 x 9.5 cm U/S, ultrasound; OB, obstetric; findings were concerning for placental abruption.

Bedside focused assessment with sonography was negative for free fluid in the abdomen or pelvis, had no evidence of pericardial effusion, and showed normal lung sliding. The fetal heart rate was calculated to be 158 bpm by ultrasound. Vaginal exam revealed a closed, long cervix with pooling of blood without active bleeding. A formal ultrasound was obtained that demonstrated a posterior placenta with an anterior 12.0 x 5.4 x 9.5 cm heterogeneous and hypoechoic mass suspicious for placental abruption (Figures 1, 2). Bedside cardiotocographic monitoring revealed a category 1 tracing. Initial laboratory workup was significant for a negative Kleihauer-Betke test, maternal blood type O+, and leukocytosis of 19.0 × 10^3^/µL (normal range: 4.3-10.8 × 10^3^/µL).

The patient was subsequently admitted to labor and delivery for observation. Management options discussed with the patient included expectant management, and induction termination due to concern for fetal hemorrhage and compromise from placental abruption. The patient opted to pursue expectant management. She was observed for evidence of progressive bleeding and disseminated intravascular coagulopathy. She developed an elevated international normalized ratio (INR) of 1.7 (normal range: 1.0-1.5) with an elevated d-dimer level, concerning for early disseminated intravascular coagulation (DIC). The initial hemoglobin dropped from 11.4 to 8.5 g/dL and hematocrit decreased from 33.9% to 25.5%. Subsequently, the patient was administered one unit of fresh frozen plasma. On her second hospital day, she developed increasing vaginal bleeding and contractions and subsequently delivered a stillborn fetus. The postnatal examination of the placenta revealed approximately 50% abruption.

## Discussion

Placental abruption is the complete or partial detachment of the placenta from the uterus and can cause both neonatal and maternal morbidity and mortality. The pathophysiology of abruption is thought to be due to the premature rupture of maternal vessels feeding the placenta. This leads to blood pooling in the decidua basalis that causes separation along the decidual-placental interface. Separation between the placenta and maternal vasculature leads to the impairment of critical placental functions such as the diffusion of nutrients and waste to and from the fetus, respectively, leading to fetal death [5-7].

Although the majority of cases of placental abruption are due to chronic placental disease, there are cases related to mechanical events such as during an MVC. The postulated mechanism of placental abruption in an MVC is stated to be a contrecoup force due to shear and tensile failure. During an MVC, rapid deceleration causes the uterus to be anteriorly displaced and creates a brief separation between the fetus and the posterior uterine wall and placenta. This generates a negative pressure between the fetus and the posterior wall that pulls the fetus backward after the anterior deceleration force decreases, a movement facilitated by the amniotic fluid. In such collisions, the maternal body may also fold over the abdomen adding to the intra-abdominal pressure. Together, these forces generate enough shear stress between the placenta and uterus to cause a placental abruption [4,8,9].

The most common risk factor for placental abruption is a previous occurrence of a placental abruption, which places the patient at a 10- to 15-fold increased risk [10]. Other risk factors include alcohol, tobacco, and cocaine use [5,6,10,11]. The prevention of injury through education on the use of seat belts and screening for abuse are considered routine components of the initial prenatal care visit. It is also important to consider domestic violence in the differential diagnosis of placental abruption, as pregnancy is a period when abuse is known to escalate [2,12]. Prenatal seat belt counseling and educational materials can effectively increase pregnant women’s knowledge of the use and placement of belts. A three-point restraint system may decrease the chance of fetal demise as a result of blunt abdominal trauma [13].

The classic presentation of placental abruption, regardless of etiology, is painful vaginal bleeding. Blood loss can be minimal to life threatening and may be concealed behind the placenta. Concealed abruptions are particularly challenging for the clinician as the patient doesn’t always have any obvious signs or symptoms of placental abruption [5]. The triad of abdominal pain, hypotension, and fetal heart rate abnormalities suggests significant placental detachment. Mild to moderate placental abruption does not cause acute laboratory findings.

Acute disseminated intravascular coagulation most often occurs when placental separation exceeds 50%. Laboratory values then demonstrate acute hemolytic anemia, elevated prothrombin time (PT), partial thromboplastin time (PTT), INR, and d-dimer level, with a decrease in the fibrinogen level. Fibrinogen levels correlate with the extent of bleeding. A fibrinogen level of less than 200 mg/dL, in the presence of placental abruption, has a 100% positive predictive value for severe hemorrhage [14]. The Kleihauer-Betke test is an unreliable predictor of placental abruption as it is positive in only a small proportion of cases [5]. Ultrasound is also a limited diagnostic tool. Immediately after placental abruption, the ultrasound exhibits the injury as echogenic amniotic fluid with retroplacental hyperechoic fluid collection, which transitions to hypoechoic in a couple of weeks after the event [15,16]. Although a placental ultrasound is a mainstay in the workup of placental abruption, only 25%-50% will be positive with 50% false negatives [5,15,17]. Contrast CT scans have a high sensitivity for the detection of placental abruption and may determine the extent of placental separation, but radiation risk to the fetus must be weighed into the decision-making process. The fetus is at the highest risk of radiation during the first 2 to 7 weeks of gestational age, during organogenesis. In the second and third trimester, the fetus is more resistant to the adverse outcomes of radiation [18]. Because imaging and laboratory studies can cause harm and are not reliable, the diagnosis of placental abruption is made clinically.

The management of placental abruption due to trauma follows standard trauma guidelines. If placental abruption is suspected, it is important to establish two large-bore intravenous access lines to rapidly stabilize the maternal cardiopulmonary system prior to the assessment of the fetus [3]. Positioning of the gravid patient during trauma assessment is unique. Uterine compression of the inferior vena cava can result in supine hypotension syndrome, especially in the third trimester. Patients should be positioned into the left lateral decubitus position to encourage optimal cardiac venous return [2]. Maternal Rh status should be evaluated in the setting of trauma, and Rhogam® should be administered when indicated. Collaboration with an obstetrician and fetal cardio-tocographic monitoring are recommended immediately following the initial trauma examination. Fetal heart rate tracing abnormalities can provide clues to the underlying condition and diagnosis. Continuous fetal heart monitoring should be performed in the setting of trauma per the established protocol [3]. The optimal length of fetal heart rate monitoring following trauma is not clear, but the majority of placental abruptions occur within 6 to 9 hours of the event. The duration of fetal monitoring should be specifically tailored to each patient, taking into consideration gestational age, pre-existing comorbidities, and mechanism of trauma. Patients with persistent abdominal tenderness, contractions, abnormal cardio-tocographic monitoring, unstable vital signs, or significant vaginal bleeding should be admitted with care coordinated between trauma surgery and obstetric teams.

## Conclusions

Placental abruption can be a disastrous complication of trauma in the gravid patient. The management of placental abruption due to trauma follows standard trauma guidelines. Continuous fetal monitoring should be specifically tailored to each patient, taking into consideration the established trauma guideline and protocols. Emergency and trauma providers must remain vigilant and diagnostically curious when assessing pregnant victims of blunt trauma to detect placental abruption and provide appropriate care to both the mother and fetus.
